# Supplementary material for: Oscillatory hyperactivity and hyperconnectivity in young APOE-ɛ4 carriers and hypoconnectivity in Alzheimer’s disease
Source: eLife. 2019 Apr 30;8:e36011. doi: 10.7554/eLife.36011 (PMC6491037; doi:10.7554/eLife.36011)
Supplement: Transparent reporting form [file elife-36011-transrepform.docx]

***eLife’s* transparent reporting form**

We encourage authors to provide detailed information *within their submission* to facilitate the interpretation and replication of experiments. Authors can upload supporting documentation to indicate the use of appropriate reporting guidelines for health-related research (see [EQUATOR Network](http://www.equator-network.org/%20)), life science research (see the [BioSharing Information Resource](https://biosharing.org/)), or the [ARRIVE guidelines](http://www.plosbiology.org/article/info:doi/10.1371/journal.pbio.1000412) for reporting work involving animal research. Where applicable, authors should refer to any relevant reporting standards documents in this form.

If you have any questions, please consult our Journal Policies and/or contact us: [editorial@elifesciences.org](mailto:editorial@elifesciences.org).

**Sample-size estimation**

- You should state whether an appropriate sample size was computed when the study was being designed
- You should state the statistical method of sample size computation and any required assumptions
- If no explicit power analysis was used, you should describe how you decided what sample (replicate) size (number) to use

Please outline where this information can be found within the submission (e.g., sections or figure legends), or explain why this information doesn’t apply to your submission:

There was no sample size calculation estimate available at the design of this study, because we are using a relatively novel methodological approach to analyze MEG data. Neuroimaging studies typically use small sample sizes of about 20 per group. We set out to dramatically increase this sample size, by recruiting more than 180 datasets, yielding more than 50-100 datasets per group for the final analysis. This is more than double a typical neuroimaging sample size for the smallest group, and unique for a neuroimaging study on young APOE carriers to date. Although a sample size computation was not performed before the design of the study, we do provide a post-hoc statistical computation of effect size (*Materials and methods, MEG data analysis*), showing a moderate effect size for the smallest group (N=51), and strong statistical power on our total group of usable data for the group comparison (N=159).

**Replicates**

- You should report how often each experiment was performed
- You should include a definition of biological versus technical replication
- The data obtained should be provided and sufficient information should be provided to indicate the number of independent biological and/or technical replicates
- If you encountered any outliers, you should describe how these were handled
- Criteria for exclusion/inclusion of data should be clearly stated
- High-throughput sequence data should be uploaded before submission, with a private link for reviewers provided (these are available from both GEO and ArrayExpress)

Please outline where this information can be found within the submission (e.g., sections or figure legends), or explain why this information doesn’t apply to your submission:

We performed our analysis once on all usable data, as described in *Materials and methods*. We set a number of inclusion and exclusion criteria for participant recruitment (*Participants*). Participants were subsequently excluded if genotyping was unsuccessful (*Participants*) or identified a number of pre-defined exclusion criteria (*APOE Genotyping*). Within the MEG data, we rejected trials of poor quality and/or containing artefacts (*MEG data analysis*). MRI data was excluded if quality was poor (*Cortical volume analysis*).

For the Alzheimer’s data, we analyzed eyes-open data only (*Alzheimer’s disease and elderly control cohort: MEG acquisition and analysis*).

**Statistical reporting**

- Statistical analysis methods should be described and justified
- Raw data should be presented in figures whenever informative to do so (typically when N per group is less than 10)
- For each experiment, you should identify the statistical tests used, exact values of N, definitions of center, methods of multiple test correction, and dispersion and precision measures (e.g., mean, median, SD, SEM, confidence intervals; and, for the major substantive results, a measure of effect size (e.g., Pearson's r, Cohen's d)
- Report exact p-values wherever possible alongside the summary statistics and 95% confidence intervals. These should be reported for all key questions and not only when the p-value is less than 0.05.

Please outline where this information can be found within the submission (e.g., sections or figure legends), or explain why this information doesn’t apply to your submission:

Details of our statistical analyses are described in the relevant sub-sections of *Materials and methods*. Numbers and other details are reported in the relevant sub-sections of the *Results* and for the behavioural and MRI data in Table 1.

(For large datasets, or papers with a very large number of statistical tests, you may upload a single table file with tests, Ns, etc., with reference to sections in the manuscript.)

**Group allocation**

- Indicate how samples were allocated into experimental groups (in the case of clinical studies, please specify allocation to treatment method); if randomization was used, please also state if restricted randomization was applied
- Indicate if masking was used during group allocation, data collection and/or data analysis

Please outline where this information can be found within the submission (e.g., sections or figure legends), or explain why this information doesn’t apply to your submission:

Samples were allocated into APOE carrier or non-carrier groups following the result of genotyping (*Materials and methods,* *APOE genotyping)*. The experimenters were thus blind to group status during recruitment and data collection, and remained so during data quality control and analysis, up until the final group comparison.

**Additional data files (“source data”)**

- We encourage you to upload relevant additional data files, such as numerical data that are represented as a graph in a figure, or as a summary table
- Where provided, these should be in the most useful format, and they can be uploaded as “Source data” files linked to a main figure or table
- Include model definition files including the full list of parameters used
- Include code used for data analysis (e.g., R, MatLab)
- Avoid stating that data files are “available upon request”

Please indicate the figures or tables for which source data files have been provided:

The ethics approval of our data precludes us from supplying the data files in a repository, but the data may be available upon request. We have explained this in a *Data availability* section. We used commercial or freely available software for MEG and MRI data analysis, with settings and details described in *Materials and methods.*
